# New Evidence and Clinical Judgment in Glaucoma

**DOI:** 10.3390/biomedicines14102311

**Published:** 2026-10-12

**Authors:** Chun Hsiung, Da-Wen Lu

**Affiliations:** 1 Department of Ophthalmology, Tri-Service General Hospital, Taipei 114202, Taiwan; drchunhsiung@gmail.com; 2 Department of Ophthalmology, College of Medicine, National Defense Medical University, Taipei 114202, Taiwan

At a glaucoma follow-up visit, a lower intraocular pressure (IOP) is reassuring. The more difficult judgment is whether the current plan will preserve useful vision over the years ahead. That judgment depends on how we interpret the patient’s measurements, how well treatment works, and what continued care asks of the patient. These are the questions that give new diagnostic and therapeutic approaches their clinical purpose.

The eight contributions to “Glaucoma: New Diagnostic and Therapeutic Approaches, 3rd Edition” address different parts of this problem. Reading them together, we are reminded how much remains to be learned between an initial observation and a dependable clinical decision. Several studies are particularly helpful in identifying where that work should begin.

One concerns the systemic information we might bring into glaucoma assessment. Yoshida et al. examined heart rate variability and acceleration plethysmography in 260 patients with primary open-angle or exfoliation glaucoma [1]. They found associations between an age-adjusted measure of comorbidity and several physiological parameters. The study draws attention to general health when interpreting these measurements, while also showing why interpretation requires care. Age is itself part of the comorbidity index, and the effects of systemic medications were not accounted for. The observed differences therefore cannot be attributed specifically to glaucoma, nor can peripheral measurements be assumed to represent optic nerve perfusion.

There is a clinical reason to pursue this line of inquiry. In a prospective cohort, Donkor et al. found that lower average 24 h mean arterial and systolic blood pressures were associated with faster visual-field loss [2]. Their work connects systemic physiology with subsequent functional change, an outcome that the cross-sectional study in this issue could not assess. It does not validate the peripheral measures studied by Yoshida et al. or establish that altering blood pressure would improve glaucoma outcomes. The next useful step would be to measure these parameters alongside ambulatory blood pressure and follow visual fields and structural imaging over time. We would want to know whether they improve prediction beyond age, IOP, and existing damage, after accounting for systemic illness and medication exposure. Only then could we consider whether they should change the intensity of follow-up or prompt further evaluation.

The treatment studies raise a related question about the evidence needed to adopt a new approach. The six-year results of the Laser in Glaucoma and Ocular Hypertension (LiGHT) trial support initial selective laser trabeculoplasty (SLT) for open-angle glaucoma and ocular hypertension, with better disease control and less need for incisional glaucoma surgery than initial eye drops [3]. This gives a clear clinical reason to investigate easier ways of delivering laser treatment. In this issue, Zollet et al. contribute a small retrospective comparison of direct SLT and conventional SLT, following 32 eyes for one year [4]. The report adds experience from routine care, but its size and design cannot establish that the procedures are equivalent.

The randomized GLAUrious trial provides an important comparison [5]. Direct SLT lowered IOP, but the trial did not meet its primary six-month noninferiority endpoint against conventional SLT. That finding leaves uncertainty about comparative efficacy, even though pressure reduction persisted through 12 months. Read alongside this trial, the issue’s report encourages further evaluation of automated delivery while reminding us to distinguish convenience from demonstrated treatment benefit. Future comparisons should follow visual-field outcomes as well as pressure and specify how retreatment and additional therapy will be handled. Treatment time and patient experience also deserve measurement. An easier procedure would be especially valuable if it made effective care more accessible without increasing the need for later intervention.

The histological study by Mukta et al. brings this discussion to the outflow pathway itself [6]. Among 95 trabeculectomy specimens, eyes exposed to ripasudil had greater total Schlemm’s canal length and open area than those without that exposure. Human tissue offers a valuable opportunity to examine the anatomy associated with clinical treatment. Yet these specimens came from eyes that had required surgery despite medical therapy, and there were no pretreatment tissue measurements. We cannot tell from this comparison whether ripasudil produced the anatomical differences or whether those differences would predict a better response to treatment.

A separate study gives that question clinical relevance. Okuda et al. reported an association between preoperative ripasudil use and greater one-year success after microhook ab interno trabeculotomy in a propensity-matched retrospective analysis [7]. Canal morphology was not measured, so the surgical findings cannot be explained by the histology reported in this issue. Nonetheless, the two observations belong in the same conversation. One describes the outflow pathway in treated human eyes, and the other suggests a possible relationship between treatment exposure and a later surgical outcome. A study connecting these observations in the same patients would be more informative than either association alone.

Such work should begin before treatment. Repeated in vivo imaging, where sufficiently reliable, together with measurements of outflow function could help determine whether anatomical change accompanies a change in IOP. A randomized study of preoperative ripasudil, followed by a standardized angle procedure, could then test the effect on surgical outcomes. The important additional question is whether an outflow measurement identifies patients who benefit more from that treatment. Answering it would bring the tissue observations closer to a decision that can be made in the clinic. Longer follow-up would still be needed to assess whether any benefit reduces subsequent medication or surgery and preserves visual function.

These studies also suggest how future research could become more useful to patients. Observational follow-up can identify whom a new approach reaches and what happens when care is interrupted. Comparative trials can test whether the approach improves outcomes under a defined treatment plan. Both should record the additional visits and interventions needed to maintain control, with consistent definitions of failure. Patients with substantial systemic illness should be included wherever feasible, because the demands of follow-up are part of the treatment decision. A favorable pressure result is easier to interpret when we also know what was required to achieve and maintain it.

As this Special Issue closes, its most useful legacy may be the questions that investigators now take forward. The systemic observations invite prospective testing of whether additional measurements improve risk assessment. The laser and tissue studies point toward comparisons that connect treatment delivery and outflow physiology with lasting clinical benefit. We hope future studies will follow these questions far enough to inform care over several years, allowing promising findings to become evidence we can use with greater confidence when a patient returns for the next visit.
